# Mapping the stabilome: a novel computational method for classifying metabolic protein stability

**DOI:** 10.1186/1752-0509-6-60

**Published:** 2012-06-08

**Authors:** Ralph Patrick, Kim-Anh Lê Cao, Melissa Davis, Bostjan Kobe, Mikael Bodén

**Affiliations:** 1 School of Chemistry and Molecular Biosciences, The University of Queensland, St Lucia, Australia; 2 Queensland Facility for Advanced Bioinformatics, The University of Queensland, St Lucia, Australia; 3 Institute for Molecular Bioscience, The University of Queensland, St Lucia, Australia; 4 Australian Infectious Diseases Research Centre, The University of Queensland, St Lucia, Australia

**Keywords:** Protein stability, Degradation, Machine learning, Post-translational modifications, Bayesian networks, Support vector machines, Prediction

## Abstract

**Background:**

The half-life of a protein is regulated by a range of system properties, including the abundance of components of the degradative machinery and protein modifiers. It is also influenced by protein-specific properties, such as a protein’s structural make-up and interaction partners. New experimental techniques coupled with powerful data integration methods now enable us to not only investigate what features govern protein stability in general, but also to build models that identify what properties determine each protein’s metabolic stability.

**Results:**

In this work we present five groups of features useful for predicting protein stability: (1) post-translational modifications, (2) domain types, (3) structural disorder, (4) the identity of a protein’s N-terminal residue and (5) amino acid sequence. We incorporate these features into a predictive model with promising accuracy. At a 20% false positive rate, the model exhibits an 80% true positive rate, outperforming the only previously proposed stability predictor. We also investigate the impact of N-terminal protein tagging as used to generate the data set, in particular the impact it may have on the measurements for secreted and transmembrane proteins; we train and test our model on a subset of the data with those proteins removed, and show that the model sustains high accuracy. Finally, we estimate system-wide metabolic stability by surveying the whole human proteome.

**Conclusions:**

We describe a variety of protein features that are significantly over- or under-represented in stable and unstable proteins, including phosphorylation, acetylation and destabilizing N-terminal residues. Bayesian networks are ideal for combining these features into a predictive model with superior accuracy and transparency compared to the only other proposed stability predictor. Furthermore, our stability predictions of the human proteome will find application in the analysis of functionally related proteins, shedding new light on regulation by protein synthesis and degradation.

## Background

Innovative proteomics technologies promise to chart protein degradation on a large scale [1-3]. The resulting data sets present an opportunity to further our understanding of metabolic protein stability through informed data analysis and the development and testing of computational models. The present study makes use of Yen and colleagues’ [1] extensive data set which measures the metabolic stability of about 8000 human proteins. We use this data set to (a) identify the underlying properties that appear to influence protein half-life, (b) develop a predictive model that integrates a number of different relevant data sets and is able to explain its predictions, (c) chart the metabolic stability of the full human proteome *in silico*, and therefore (d) infer what features influence stability on a global scale.

High-throughput methods for measuring protein degradation typically involve either metabolic labeling or protein tagging. Of the four largest data sets currently available, three were generated in human cells, one using labeling [2], and two using a tagging approach [1,3], while the fourth data set was generated using protein tagging in yeast [4].

Doherty and colleagues used stable isotope labeling with amino acids in cell culture (SILAC) coupled with mass spectrometry, (MS) to measure the degradation rate of 576 proteins [2]. Cells were grown in medium containing ^13^C_6_arginine before being transferred to ^12^C_6_arginine medium. MS was used to measure the shrinking abundances of ^13^C_6_ labeled proteins over several time points and a degradation rate was calculated by fitting the abundances to a single exponential curve. One advantage of this method is that metabolic labeling causes minimal cell perturbation. However, by nature of using mass spectrometry, the measurements will be biased to highly abundant, and theoretically more stable proteins [5].

Belle and colleagues used tandem affinity purification (TAP) tagging along with cycloheximide inhibition of transcription and western blotting to measure the half-life of over 3000 yeast proteins [4]. The most recent tag-based method is called “bleach chase” and was used to measure the half-life of 100 proteins from H1229 cells [3]. Proteins were tagged with yellow fluorescent protein and their half-lives were inferred from “bleaching” some cells with a pulse of light and measuring the difference in fluorescent decay between bleached and non-bleached cells.

Another tag-based approach implemented in human HEK293T cells used a dual-fluorescent tagging method called global protein stability profiling (GPSP) [1]. The GPSP method uses two fluorescent proteins, enhanced green fluorescent protein (EGFP) and *Discosoma* sp. red (DsRed), which are expressed on a single mRNA transcript. The DsRed protein acts as a control, while EGFP is expressed as an N-terminal fusion with a protein of interest. Coupling this approach with fluorescence activated cell sorting (FACS) and microarray analysis, the authors were able to measure the stability of approximately 8000 human proteins, and it is this data set we use in our study.

An important consideration of N-terminal fusion is the interference that the EGFP tag could have on the function of N-terminal signal sequences. A recent review on the use of fluorescent protein tagging points out that approximately one third of human protein-coding genes contain position-dependent sequence information [6]. In the case of proteins with N-terminal signal peptides, or signal anchors, the fusion of a fluorescent protein to the N-terminus is likely to interfere with normal localization. Indeed, Yen and colleagues [1] found that unstable proteins contained an enrichment of membrane protein gene ontology (GO) terms but remark that it is unclear what effect fluorescent tagging will have upon the measurement of global degradation rates.

Huang and colleagues recently explored a range of predictive features in the GPSP data set and indicated that a simple associative model can classify protein stability with a reasonable accuracy – as evaluated using the same data set [7]. However, without paying attention to the potential bias caused by N-terminal tagging, a computational model may contain the same biases. Therefore, our paper presents a protein stability model based on the largest of the present protein degradation data sets with emphasis on minimising experimental bias. Indeed, it may be possible to discount the influence of experimental artefacts by first exploring and understanding their impact on models.

We created a method for classifying proteins as having a high metabolic stability (i.e. long half-life) or low stability. We developed this method using the GPSP stability data set, which is by far the most extensive available, and thus easiest to cross-reference to other complementary data resources. We considered that this data set may contain a bias portraying proteins with N-terminal signal peptides and anchors as metabolically unstable due to interference caused by the experimental technique. Consequently, we developed and tested models on two sets of proteins: a full set, and a trimmed set with secreted and transmembrane proteins removed.

Using complementary resources, including the Human Protein Reference Database (HPRD), a wide range of predictive features were explored. We identified groups of features that are statistically enriched in both stable and unstable proteins, ultimately to understand if they may be used to infer metabolic stability levels. We subsequently designed a model that explicitly recognizes and integrates known factors of the relevant processes and employed machine learning to optimise its ability to generalize to novel proteins. Finally, to illustrate metabolic stability on a system scale, we used the model to score the stability of all proteins contained in the HPRD.

### Features relevant to protein stability

Protein degradation via the proteasome is mediated through poly-ubiquitination [8]. However, there are a number of other post-translational modifications (PTMs) for which a role in either targeting proteins to or protecting proteins from the degradative machinery has been suggested. These modifications include phosphorylation, prolyl hydroxylation, glycosylation and small ubiquitin-related modifier (SUMO) conjugation [9]. For example, phosphorylation can either promote or inhibit ubiquitination by regulating the E3 ligases responsible for ubiquitination [10]. Glycosylation is used as a form of protein quality control in the endoplasmic reticulum, the folding location of most secreted and integral membrane proteins [11], and this modification can act as a signal for misfolded proteins to be translocated to the cytosol for degradation.

A variety of features have been proposed to influence the targeting of a protein to the ubiquitin proteasome system. The N-end rule is one of the best documented examples of sequence-based degradation signals [12]. The rule originally stated that the *in vivo* half-life of a protein is associated with the identity of its N-terminal residue, causing high levels of binding selectivity for the E3 ligases that target proteins for ubiquitin-mediated degradation. The rule classified N-terminal residues as stabilizing, or belonging to one of three classes of destabilizing residues: primary, secondary or tertiary. Recently, the rule was extended when it was discovered that N-terminal acetylation of most amino acids acts to create N-degrons [13]. Now it is believed that all but two amino acids (glycine and proline) can act as degradation signals upon acetylation, and are therefore considered “destabilizing”. However, the authors note that many proteins may still have a high level of metabolic stability despite the presence of an N-degron. It is possible that long-lived proteins are protected from degradation through complex formation or folding that makes the N-degron inaccessible.

It has been suggested that structural disorder is correlated with protein stability [14]. A bioinformatic study on yeast data found that structural disorder had a weak, but significant inverse correlation with protein half-life [14]. However, a separate study of protein structural disorder found that highly disordered proteins (where high disorder is defined as a protein with greater that 80% sequence disorder) had far greater metabolic stability than proteins with low structural disorder [15].

A correlation between the frequency of certain amino acids has also been reported[1,7]. One study demonstrated that the frequencies of tryptophan, cysteine, leucine and threonine were negatively correlated with protein stability, and conversely, that glutamic acid, aspartic acid, lysine and asparagine were positively correlated with protein stability [1]. The exact biological mechanisms underlying the relationship between amino acid composition and stability are unknown, though there are likely to be a variety of factors. For example, the PEST hypothesis claims that short hydrophilic sequence segments enriched in certain residues are correlated with protein instability [16]. What mechanisms are behind this are unknown, and it has also been suggested that PEST regions are actually sequence areas enriched in amino acids that confer phosphorylation modification sites [10].

### Previous work on modeling protein stability

There are a number of ways to define metabolic stability. For example, one study presented a probabilistic method for classifying the metabolic stability *in vitro* of chemical compounds [17]. However, to the best of our knowledge there is currently only one published predictor for intracellular protein stability [7]. The contribution of this published work is two-fold: Huang and colleagues identified optimized sets of features relevant to protein stability, and created a predictor that classifies protein stability based upon the “best” feature vectors. Using the Yen [1] data set, the authors created four classes of protein stability (based on a discrete measurement called the protein stability index, or PSI): short (PSI < 2), medium (2 ≤ PSI ≤ 3), long (3 ≤ PSI ≤ 4) and extra long (PSI ≥ 4). The authors went on to define a list of 376 possible feature components that are believed to contribute to protein stability. These included various biochemical/physiochemical attributes of proteins (such as amino acid composition, hydrophobicity and polarity), protein subcellular locations, KEGG enrichment scores, and the number of complexes a protein is involved in.

To determine what features distinguish between different classes of stability, the authors defined three 2-class problems based on their four stability classes: (1) short and medium *vs* long and extra long, (2) short *vs* medium and (3) long *vs* extra long. For each problem, they ordered the feature vectors according to the maximum relevance and minimum redundancy method, which ranks elements in a vector according to their relevance to a target, and redundancy against each other [18]. To optimise the elements in the feature vectors, they used incremental feature selection (IFS) with nearest neighbor (NN) (using 1-cosine distance as the metric) to classify proteins based on increasing feature vector sizes. For each vector size, they used jack-knife cross-validation to determine the accuracy of NN. For problem (1), they reported an overall accuracy of 72.8% using 62 features; for problem (2), an accuracy of 69.8% with 43 features, and for problem (3), an accuracy of 67.8% with 122 features. On a cautionary note, the automated IFS scheme is likely to invoke a “selection bias”, whereby features are optimal for that particular data set, leading to inflated performance figures [19].

The authors reported that localisation to the cell membrane was an important contributor to predicting protein stability. In our own analysis on the set of proteins used in their study, we found that proteins localised to the membrane or the cell surface were significantly over-represented in the “short” class compared to other proteins (Additional file 1: Data Set 1). Furthermore, the most significant feature for problem (1) according to the optimal feature vector is the frequency of hydrophobic residues. We found that an average of 35% of residues in the “short” class proteins were classified as hydrophobic, compared to only 29% in the “extra long” class. Considering the hydrophobic nature of transmembrane domains, this is likely a reflection of the “short” class proteins localised to the membrane. If the GPSP data is indeed biased towards membrane proteins, then it appears that the NN model shares that bias.

The NN method as employed by Huang and colleagues [7] simply classifies a query protein based on the class of the “nearest” protein. As a result, this method does not give us information on how given features influence stability – whether they correlate with stable or unstable proteins. However, the use of probabilistic machine learning methods such as Bayesian networks can give a transparent and explainable model of protein stability. Though Huang and colleagues have presented the first attempt at computationally modeling protein stability, this work can be improved through the removal of potential experimental bias from the data, and the use of a computational method that can explain its predictions.

### Bayesian networks

We chose to use Bayesian networks to model metabolic stability and the relationships between relevant features for several reasons. First, a Bayesian network can represent a large number of different types of features to influence the outcome, recognizing only dependencies that we believe exist. Second, by virtue of its probabilistic nature, uncertain observations can be incorporated, missing data can be managed, and flexible queries to probe and explain predictions can be entertained [20,21]. Finally, though most observations are strictly “Boolean” (true or false), we are able to integrate “continuous” scores produced by methods particularly suited to challenging though largely independent sub-problems, such as support-vector machines (SVMs) applied to detect protein sequence similarity and position-weight matrices (PWMs) applied to recognize PTM sites.

We represent observations about stability, presence of specific domains, sequence and structural features, PTMs etc as random variables *X*_1_*X*_2_,…,*X*_*N*_, that may take values *x*_1_*x*_2_,…,*x*_*N*_. Variables are organized “graphically” into a network, with *pa*(*X*) representing the set of “parent” variables of *X*, thereby identifying what dependencies can be captured. The joint probability of all variables is given by

(1) $$P(X_{1}=x_{1},\ldots ,X_{N}=x_{N})=\overset{N}{\underset{i=1}{\prod }}P(X_{i}=x_{i}|\mathrm{pa}(X_{i}))$$

We discuss the selection of relevant variables and their relationships in Section “Data resources and feature identification”, including the use of latent (un-observed) variables. Parameters are set by using the expectation-maximization (EM) algorithm on a training set [22]. We also separate out data used to parameterize PWMs to score PTM sites and data used to train SVMs equipped with the 1-spectrum kernel to map a protein sequence to its stability class [23].

## Results and discussion

We have described the three most large scale protein stability data sets available in human cell lines. However, each study has applied different technology to different cell types, with different units for measurement for protein degradation. A comparison of the data generated by the GPSP method [1] and the metabolic labeling/mass spectrometry method [2] showed very little correlation between the two data sets [24]. Furthermore, our own comparison of these data sets (summarized in Table 1) shows that there is no correlation between any of the three major data sets. We hypothesized that the potential bias caused by N-terminal tagging in the GPSP data could be resulting in the disparity, but removing secreted and transmembrane proteins did not improve the correlation (Table 1). Disparity amongst results does not necessarily mean experimental error, but could be a result of differing cell types, tuning a protein’s lifespan to the individual requirements of the cell. Regardless of the causes behind the variability, an unfortunate side-effect is that integrating and analyzing data from multiple sources becomes much more difficult. Therefore, the model presented in this study is based singularly upon the GPSP data generated by Yen and colleagues [1].

**Table 1:** Comparison of protein degradation data sets


**Data set comparison**	**P-value (Spearman rank correlation)**	**Adjusted *ρ* **^**2**^
GPSP *vs* SILAC	0.976	-0.002305
GPSP *vs* SILAC (non-sec/TM)	0.7525	-0.003241
GPSP *vs* Bleach Chase	0.4642	-0.008504
SILAC *vs* Bleach Chase	0.3826	-0.01887

Comparisons were made between the Global Protein Stability Profiling method (GPSP) and the Stable Isotope Labeling of Amino Acids in Culture (SILAC) method. This comparison was repeated with transmembrane and secreted proteins removed. The bleach chase data was also compared with GPSP and SILAC. All comparisons were made with a Spearman rank correlation, as well as being fitted to a linear model.

### Identifying stability groups of proteins

**Figure 1:**
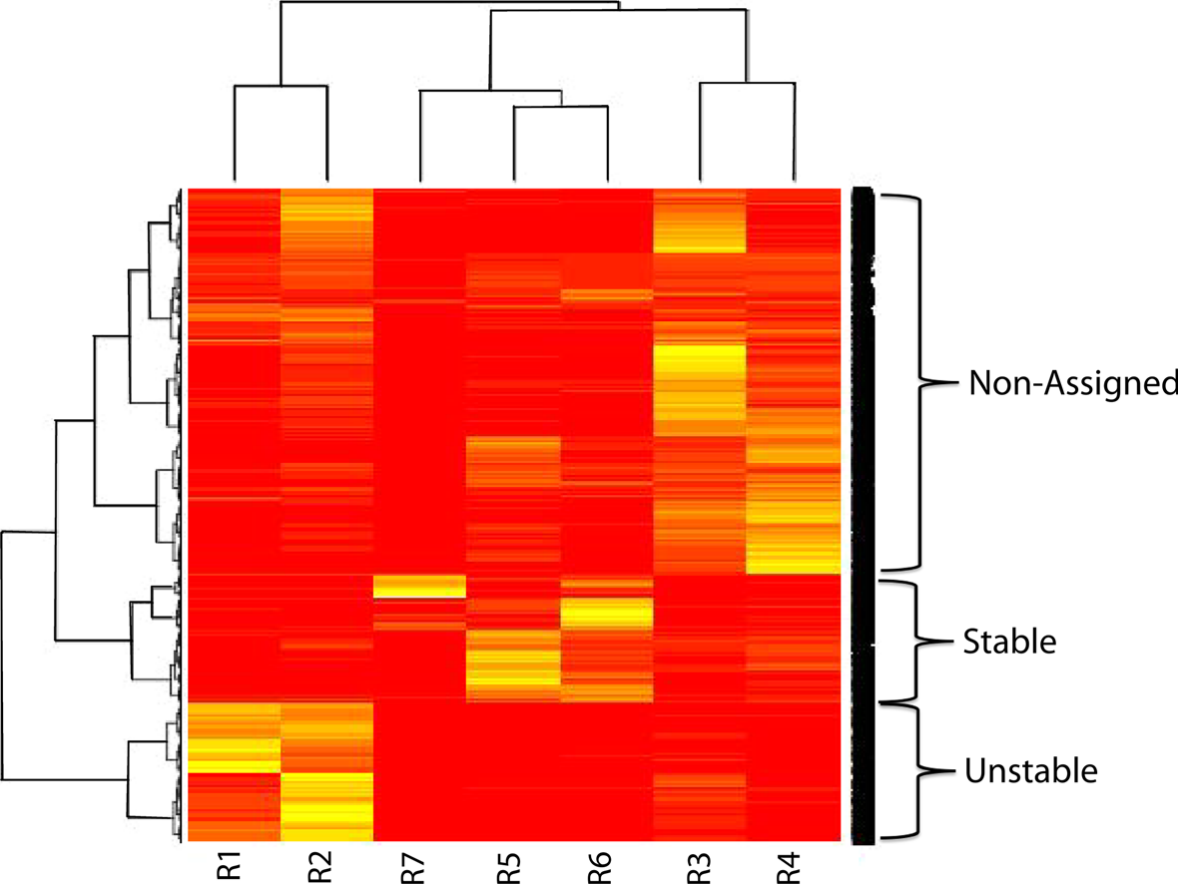
**Heat-map and cluster analysis generated from the output of the Yen experiment [**[1]**].** A Euclidean distance metric was used for the cluster analysis. Bright (yellow) colouring in the heat-map represents a value approaching 1, with values approaching 0 having a dull (red) colouring. Labels on the right show how the data can naturally be broken up into groups of stable and unstable proteins, with the remainder being classed as “non-assigned”.

The authors of the GPSP data divide cells into seven sub-populations based on increasing ratios of EGFP/DsRed as defined during FACS (R1 – R7). The distribution of cells across these R values (with the distributions summing to 1) was used to infer the stability of the EGFP-fusion protein they expressed. The authors defined a weighted mean, the Protein Stability Index (PSI) for representing this information as a continuous variable. The PSI is calculated by:

(2) $$\mathrm{PSI}=\overset{7}{\underset{i=1}{\sum }}R_{i}*i,$$

where *R*_*i*_ is the proportion of cells in bin *i* for a given gene. This metric for stability classification was also used by Huang and colleagues [7], as well as other studies that have employed the GPSP data [15,25]. However, the selection of PSI classification thresholds is non-trivial. Therefore, instead of using the PSI, we first explored how proteins grouped based on the original weight distributions across the 7 bins (using Euclidean distances between data points). Figure 1 shows a cluster dendrogram and a heat-map highlighting the R values that the proteins are enriched in. We noted in particular two distinct groups representing the extremes of protein stability. These two groups seemed a natural choice for investigating what features influence stability, as well as constructing a binary classifier. The bottom group, henceforth labelled “unstable”, contains proteins enriched in the highly unstable R1 and R2 bins. The “stable” group contains proteins that are enriched in the stable R5, R6 and R7 bins. The remainder were classified as “non-assigned”. Approximately 20% of the proteins fell into the unstable class, another 20% into the stable class, and the final 60% fell into the non-assigned class.

### Identifying features correlated with stability

We investigated five types of features believed to be related to protein degradation: (1) PTMs, (2) domain and architecture types, (3) N-terminal residues, (4) structural disorder and (5) amino acid composition. For each feature (with the exception of amino acid composition) we used Fisher’s Exact test (corrected for multiple testing) to determine whether there was a significant over- or under-representation in the stable or unstable protein classes, relative all other proteins. The results from the statistical analyses on proteins in the unstable class are summarized in Table 2, and Table 3 contains the results for proteins assigned to the stable class. Phosphorylation and acetylation modifications were both over-represented in stable proteins while being under-represented in unstable proteins: 72% of stable proteins were phosphorylated compared to 30% of unstable proteins, and 36% of stable proteins were acetylated compared to only 4% of unstable proteins. The opposite trend was seen in glycosylation, where only 0.3% of stable proteins were glycosylated, compared to 6% of unstable proteins.

**Table 2:** Feature analysis of proteins in unstable class


**Unstable *vs* Stable Plus Non-Assigned**	
**Domain/Motif**	**E-value**
Transmembrane	3.75e-96 ‡
Signal Peptide	9.15e-50 ‡
Loops/Coils	1.36e-06
Immunoglobulin	1.77e-04
Immunoglobulin Like	2.01e-03 ‡
Immunoglobulin C	4.286e-03 ‡
Zinc Finger C2	1.73e-02
Cadherin	2.39e-02 ‡
**PTM**	
Phosphorylation	2.29e-15
Acetylation	6.15e-13
Glycosylation	9.73e-04 ‡
**Disorder**	
Hot loops: 0-20%	8.77e-03 ‡
Loops/Coils: 20-40%	1.06e-03
Hot loops: 20-40%	9.11e-06
Rem465: 20-40%	3.41e-05
**N-degron**	4.86e-80 ‡

Feature types found to be significantly (Fisher’s Exact test, E-value < 0.05) over-/under-represented in the unstable protein class when compared to stable and non-assigned proteins. E-values for over-represented features are indicated with the symbol ‡, otherwise the E-value represents an under-representation. Features include domain/architecture types, PTMs, and disorder classifications based on three disorder types (loops/coils, hotloops and rem465) and percentage of sequence containing the disorder type. Lastly, the presence of a destabilizing N-terminal residue is shown, defined as the N-terminal residue of a mature protein being one of R, K, H, F, L, W, I or Y. These results are from the full data set (without removal of transmembrane or secreted proteins).

**Table 3:** Feature analysis of proteins in stable class


**Stable *vs* Unstable Plus Non-Assigned**	
**Domain/Motif**	**E-value**
Transmembrane	3.99e-22
Signal Peptide	3.39e-17
RNA Recognition Motif	1.20e-03‡
**PTM**	
Acetylation	4.11e-28‡
Phosphorylation	1.56e-21‡
Glycosylation	5.65e-03
**Disorder**	
Hot loops: 0-20%	5.72e-03‡
Coils: 80-100%	1.56e-02‡
Hot loops: 80-100%	4.72e-03‡
**N-degron**	5.11e-32

Feature types found to be significantly (Fisher’s Exact test, E-value < 0.05) over-/under-represented in the stable class when compared to unstable and non-assigned proteins. E-values for over-represented features are indicated with the symbol *‡*, otherwise the E-value represents an under-representation. Features include domain/architecture types, PTMs, and disorder classifications based on three disorder types (“loops/coils”, “hotloops” and “rem465” as defined by Linding and colleagues [26]) and percentage of sequence containing the disorder type. Lastly, the presence of a destabilizing N-terminal residue is shown, defined as the N-terminal residue of a mature protein being one of R, K, H, F, L, W, I or Y. These results are from the full data set (without removal of transmembrane or secreted proteins).

The most significant outcomes of the domain and architecture analysis was the strong over-representation of transmembrane domains and signal peptides in the set of unstable proteins. These were over-represented in unstable proteins while being under-represented in stable proteins: 63% of unstable proteins contained transmembrane domains compared to 4% of stable proteins. These findings were supported by an analysis of GO terms, which also showed that unstable proteins were significantly more likely to contain a transmembrane domain than were proteins from the stable or non-assigned class. Signal peptides were present in 44% of unstable proteins compared to 3% of stable proteins.

The results for the N-terminal residue analysis were highly significant, with destabilizing N-terminal residues (“destabilizing” being defined according to the original N-end rule [12]) being over-represented in unstable proteins while being under-represented in stable proteins. We found that approximately 50% of unstable proteins had a “destabilizing” N-terminal residue compared with a far lower proportion (under 10%) in the remaining proteins. The opposite was seen in the stable class, where under 2% of proteins contained destabilizing N-terminal residues.

We grouped proteins according to their structural disorder using the disEMBL software (http://dis.embl.de/html/download.html) referring to the types used previously by Linding and colleagues [26]. The disorder types are defined as “loops/coils” (secondary structures that can serve as conditions for structural disorder), “hot loops” (a subset of loops/coils with high mobility and higher disorder propensity) and “rem465” (remark465 entries in PDB indicating missing coordinates in X-Ray structure). A total of 15 classifications were used, based on the 3 disorder types as defined by Linding and colleagues [26], and 5 levels of disorder as used in an earlier study [15]. Levels were classed according to the percentage of a sequence containing disorded residues of a given type. The levels were: (1) ≥ 0% and ≤ 20% of sequence containing disordered residues, (2) > 20% and ≤ 40%, (3) > 40% and ≤ 60%, (4) > 60% and ≤ 80 %, (5) > 80% and ≤ 100%.

There were a number of disorder types found to be statistically significant. Tables 2 and 3 show that in both stable and unstable classes there was an over-representation of the very low level disorder “hot loops: 0-20%” class. This disorder type was present in 44% of unstable proteins, and present in about 50% of stable proteins. The low level 20-40% disorder class for all disorder types were over-represented in the unstable protein class. The highly disordered class of 80-100% hot loops and Loops/Coils was over-represented in the stable set of proteins.

### A predictive model of protein stability

**Figure 2:**
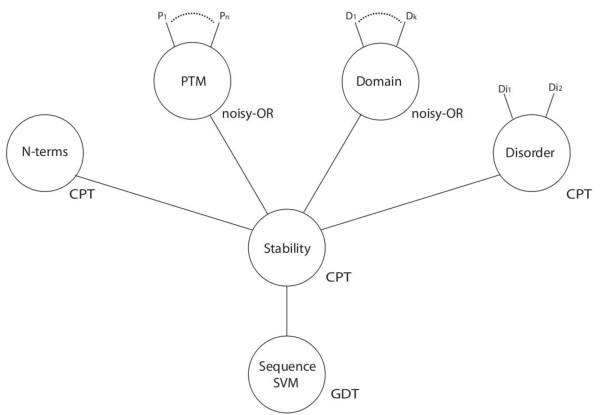
**Graph representing BN + SVM model.** The type of model parameters are indicated by conditional probability tables (CPT), noisy-OR (conditional probability table with a noisy-OR assumption) and Gaussian density tables (GDT) representing continuous values. For the sake of clarity, two continuous nodes that are children to the tyrosine and serine/threonine phosphorylation PTM nodes are not included in the graph. These continuous nodes contain the PWM scores for the sequence. The BN model is identical, but with “Sequence SVM” removed. The SVM model contained only the “Stability” node with the “Sequence SVM” node as a child.

We are interested in resolving what features have a causal relationship with the classification of metabolic stability. We are also interested in establishing dependencies between features relevant to stability. Both these issues can be addressed using machine learning models. We tested the ability of a strawman SVM trained on sequence data (we refer to this model as “SVM”), a BN integrating the aforementioned features (Tables 2 and 3) excluding sequence features (we refer to this model as “BN”), and a complete model (henceforth “BN+SVM”; Figure 2), to classify proteins into stable and unstable classes. The complete model incorporates the SVM output as an additional continuous variable.

**Figure 3:**
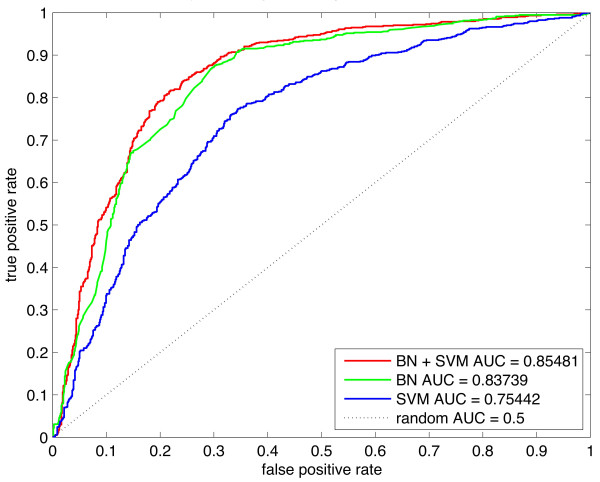
**Comparison of true positive and false positive rates for protein stability prediction models.** Receiver operating characteristic (ROC) curves for the BN, SVM and BN+SVM models were calculated using 10-fold cross validation. We evaluated the three models on a total of 743 unstable proteins and 794 stable proteins.

We evaluated model performance through receiver operating characteristic (ROC) analysis and calculating the area under the ROC curve (AUC) [27]. We evaluated the performance of three versions of the model: SVM, BN, and BN+SVM. Models were first evaluated on a data set made up of 743 proteins from the stable protein set and 794 proteins from the unstable protein set using ten-fold cross-validation over five different data set splits. Figure 3 shows the performance of the SVM, BN and BN+SVM models for this data set. All three models have AUCs well above random (0.5), and the BN+SVM model performs best. These results are consistent over multiple cross-validation runs with different data set splits. The mean AUC of the BN+SVM model was 0.85 with a standard deviation of 0.0026. The mean AUC of the BN model was 0.84 with a standard deviation of 0.0012, and the mean AUC of the SVM model was 0.76 with a standard deviation of 0.0043. To measure the performance of the models using a threshold, we took the threshold that occurs at the maximum F-score and calculated sensitivity, specificity and Matthews correlation coefficient (MCC). These results including the AUCs are summarised in Table 4.

**Table 4:** Summary of performance metrics for each of the trained models


**Model**	**AUC**		**F-score**		**MCC**		**Sensitivity**		**Specificity**	
	** *μ* **	** *σ* **	** *μ* **	** *σ* **	** *μ* **	** *σ* **	** *μ* **	** *σ* **	** *μ* **	** *σ* **
BN+SVM(1)	0.85	0.0026	0.8	0.002	0.58	0.01	0.9	0.031	0.67	0.045
BN(1)	0.84	0.0012	0.8	0.0002	0.58	0.0007	0.9	0.005	0.66	0.0076
SVM(1)	0.74	0.0043	0.73	0.004	0.42	0.014	0.83	0.027	0.58	0.044
BN+SVM(2)	0.75	0.0052	0.77	0.009	0.39	0.035	0.88	0.032	0.46	0.072
BN(2)	0.66	0.0046	0.73	0.001	0.29	0.07	0.82	0.08	0.45	0.18
SVM(2)	0.73	0.0041	0.76	0.004	0.35	0.03	0.85	0.039	0.46	0.08

Various performance metrics for each of the models trained on the full data set, as well as the trimmed data set. For example, BN+SVM(1) refers to the BN+SVM model trained on the full data set, while BN+SVM(2) refers to the same model trained on the trimmed data set. For each model we present the area under the curve (AUC) for a receiver operating characteristic analysis, and find the maximum F-score. The threshold from the maximum F-score was also used to calculate Matthews correlation coefficient (MCC) as well as sensitivity and specificity. For each metric the mean (*μ*) and standard deviation (*σ*) is shown for five cross-validation runs.

**Figure 4:**
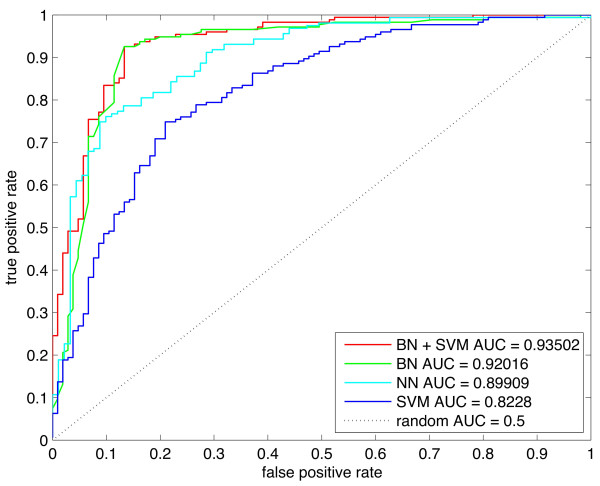
**Comparison with the IFS and NN classifier.** The performance of the BN, SVM and BN+SVM models were compared against Huang and Colleagues’ [7] IFS plus NN method through calculation of ROC. The models were evaluated on a subset of 250 genes overlapping between our stable/unstable classes and the extra long/short classes as defined by Huang and colleagues [7].

We also did a performance comparison with Huang and colleagues’ [7] IFS and NN model (see Methods for an explanation of the comparison) in order to determine which method classifies protein stability most accurately. We took a subset of proteins that overlapped with their “extra long” class and our stable class, as well as a subset of proteins that overlapped with their “short” class and our unstable class. Evaluating the NN model on this subset gave an AUC of 0.899 (Figure 4). We also evaluated the performance of our models on this data, and found that the BN+SVM and BN models both had superior performance to the NN model with AUCs of 0.935 and 0.92, respectively (Figure 4)

**Figure 5:**
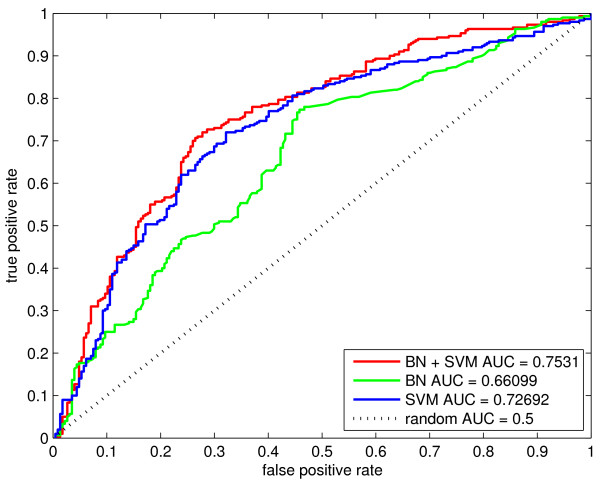
**Comparison of protein stability prediction models trained on a trimmed data set.** The performance of the BN, SVM and BN+SVM model on a trimmed data set with secreted and transmembrane proteins removed. Due to the set containing a smaller number of samples (300 stable proteins and 227 unstable proteins), 25-fold cross validation was used to calculate the ROC curves.

We created a trimmed data set with transmembrane/secreted proteins removed to test the models on proteins not affected by N-terminal fluorescent tagging. Re-visiting the statistical analysis on this data set, we found that transmembrane domains, signal peptides and glycosylation were no longer over-represented in unstable proteins. We evaluated the performance of the three models with twenty five-fold cross validation due to a smaller number of observations. Figure 5 shows ROC curves for the 3 models trained and tested on the set that was cleansed from proteins that are potentially subject to experimental bias. All three models perform worse on the smaller data set, though performance is still well above random. The BN+SVM model had an average AUC of 0.75 with a standard deviation of 0.0052. The BN model performed worse with an average AUC of 0.66 and a standard deviation of 0.0046. In contrast to the results obtained using the original data set, the SVM model performed better than BN with an average AUC of 0.73 and a standard deviation of 0.0041. For each model we also calculated the sensitivity, specificity and MCC that occurs at the threshold corresponding to the maximum F-score (Table 4).

### Classifying the stability of all human proteins

There are several uses for a global prediction of protein stability values. First, the predictions allow us to ascertain how well the model generalizes. Secondly, we can measure the stability of all proteins through estimating how many proteins are classified as stable or unstable. Finally, we can investigate what features are correlated with stability on a global scale, and compare the findings with experimental data.

We used the BN+SVM model to predict the stability of 30,019 protein isoforms catalogued in HPRD. Two predictions were made, one with the BN+SVM model trained on the full data set (prediction 1, or P1), and one with the BN+SVM model trained on the trimmed data set (P2). Additional file 2: Figure S1 shows a density plot of P1, and Additional file 3: Figure S2 shows a density plot of P2. As the BN+SVM model produces a probability representing the belief that the protein is stable, thresholds are required to classify protein predictions into stable and unstable groups. The thresholds can be modified according to a desired level of true positives / false positives, and are most likely be different between P1 and P2. For our analysis of P1, an unstable protein was defined as having a score less than 0.2, while a stable protein scored greater than 0.75. This resulted in 29% of proteins being assigned to an unstable class, and 26% to a stable class (Table 5). When applying these thresholds to proteins from the GPSP training set, 60% of unstable proteins (as according to the cluster analysis) are correctly classified as unstable, and 42% of stable proteins are correctly classified as stable.

**Table 5:** Breakdown of numbers of proteins across stability groups for experimental and predicted classes


**Data set**	**Unstable**	**Non-assigned**	**Stable**
Training/testing data	795	2442	743
Predictions 1 (P1)	6974	10683	6357
Predictions 2 (P2)	3319	17475	3220

The first row shows the number of proteins in each of the stable, unstable and non-assigned groups that were allotted based on the cluster analysis of the GPSP data [1]. This data was employed in all of the statistical analyses and the proteins in the stable and unstable classes were used for training and testing the models. The second and third rows show how the predictions made by the BN+SVM model trained on the full data set (P1) and the predictions made by the BN+SVM model trained on the trimmed data set (P2) can be assigned to stability classes when using thresholds to define predicted “stable” and “unstable” proteins. For P1, a stable protein was defined as scoring above 0.75 and an unstable protein had to score below 0.2. For P2, a stable protein was required to score above 0.7 and an unstable protein to score below 0.3. For both P1 and P2, if a protein scored above the “unstable” threshold and below the “stable” threshold it was classified as “non-assigned”. These thresholds were set such that the number of proteins predicted to fall into the three different stability groups reflected the numbers from the cluster analysis on the GPSP data.

Given these predicted stability groups we again tested for the presence of PTMs and domain/architecture types. A full catalogue of statistically significant features is contained in Additional file 4: Data Set 2 (domains) and Additional file 5: Data Set 3 (PTMs). Apart from the features already noted (Table 3), PTMs including methylation, S-nitrosylation and sumoylation were over-represented in stable proteins, and under-represented in unstable proteins. A number of new domain and architecture types were found as well. For example, nuclear localization signals (NLS), nuclear export signals, (NES) and other nuclear related domains were over-represented in stable proteins and under-represented in unstable proteins. A similar result was found by Yen and colleagues [1], who noted that stable proteins were enriched in nuclear GO terms.

For P2, proteins scoring less than 0.3 were assigned to the unstable class and those scoring greater than 0.7 were assigned to the stable class. 14% of proteins fell into the unstable class, and 13% were assigned to the stable class. We found that signal peptides were under-represented in both stable and unstable proteins. Transmembrane domains were under-represented in stable proteins, and though they were over-represented in unstable proteins, the significance level was greatly reduced to what was seen in P1 where we saw a highly significant over-representation of secreted and transmembrane proteins in the unstable class. It appears that when trained on the trimmed data set, the model is scoring secreted and transmembrane proteins such that they are largely falling into the “non-assigned” classification – an expected outcome given the lack of those proteins in the training data. This is supported by an under-representation of glycosylation modifications in the unstable class. These results indicate that when trained on the trimmed data set, the model is no longer making the counterintuitive decisions that may be a result of N-terminal tagging interference with protein localisation.

One useful feature of stability values is the ability to estimate the effect of global stability on specific groups of proteins. To that end, we investigated the global stability of signaling proteins using a list of approximately 6000 proteins annotated with the biological process GO term “signaling”. We found that signaling proteins were highly over-represented in the P1 unstable class (Fisher’s exact test, P = 3.08e-29). There was also a smaller, though still significant over-representation of signaling proteins in the original unstable class determined by cluster analysis (Fisher’s exact test, P = 0.019). However, the opposite was seen in P2 where signaling proteins were significantly under-represented in the unstable class (Fisher’ exact test, P = 2.54e-14) and significantly over-represented in the stable class (Fisher’s exact test, P = 1.34e-16). We also noted several domain types such as SH2 and SH3 known to be involved in signal transduction that were over-represented in both the P1 and P2 stable classes [28].

In summary, the two predicted sets of stability values have some shared, and some distinct features that govern whether a protein might be classified as stable or unstable. We have made the predicted stability values for P1 and P2 available in Additional file 6: Data Set 4, as well as SVM scores for all proteins (Additional file 7: Data Set 5). The Supplementary Material also contains the feature vectors that were used for training/testing the models and generating the predicted values (Additional file 8: Data Set 6, Additional file 9: Data Set 7 and Additional file 10: Data Set 8). While the stability values for P1 are reliable predictions for intracellular proteins without N-terminal signaling sequences, caution should be exercised when considering secreted and transmembrane proteins.

## Discussion

We have presented a model for classifying metabolic protein stability that combines five groups of features: (1) post-translational modifications, (2) domain types, (3) structural disorder, (4) the identity of a protein’s N-terminal residue and (5) amino acid sequence. The ability of the model to predict a protein’s stability is high, with an AUC of 0.85 for the BN+SVM model. Furthermore, at a low false positive rate of 20% the model exhibits a high true positive rate of 80%. We compared our approach with the IFS and NN method employed previously [7] and demonstrated the superiority of our model. When evaluating our models on the subset of data used for comparison with the NN model, we found that at a 20% false positive rate, the BN+SVM model performs with a true positive prediction rate of approximately 95%. Meanwhile the NN model has just over an 80% true positive rate.

Despite the improved performance, the BN+SVM model relies on far fewer features than the NN model – 19 compared to 62 for the “short/medium vs long/extra long” classifier. This is an indicator that the features we have chosen for the BN+SVM model have far more powerful predictive capabilities than those used by Huang and colleagues [7]. Furthermore the IFS method, while it can generate optimised vectors of features, cannot say how the features relate to protein stability. In contrast, we have found that post translational modifications such as phosphorylation and acetylation are significantly over-represented in stable proteins, that proteins with 80 – 100% structural disorder are also over-represented in stable proteins and that destabilizing N-terminal residues are over-represented in unstable proteins.

It must be acknowledged that there are potentially detrimental effects that fluorescent tagging could have on global protein stability measurements for some proteins, which could bias computational models. The results from our feature analysis are consistent with the hypothesis that fluorescent tagging of co-translationally translocated proteins (those possessing a signal peptide or transmembrane domain) causes unnaturally quick degradation. The transmembrane domain was highly over-represented in unstable proteins with an E-value of 3.75e-96 – a far more significant number than any other feature. This was consistent with a GO term analysis, which found membrane GO terms over-represented in unstable proteins. It has been noted before that there exist a class of short-lived transmembrane proteins that are degraded by the proteasome [29], but there is no reason to think that transmembrane proteins are unstable in general. It is known that N-terminal fluorescent tagging of proteins with N-terminal signal peptides can interfere with correct localisation [6], and also that protein quality control mechanisms can rapidly degrade misfolded proteins [9].

In light of potential experimental bias, we have tested our model on non-secretory and non-transmembrane proteins, and have found that it continues to perform well with an AUC of 0.75. Even though the model has decreased performance accuracy on the trimmed data set, the fact that the performance level remains high shows that Bayesian networks are powerful tools for computationally modeling metabolic protein stability. Furthermore, it is no longer making the unlikely inference that all membrane and secreted proteins are unstable.

While the models are quite successful at classifying proteins into the “extreme” groups of stable and unstable, there is a long way to go in understanding the individual determinants of protein degradation. Consider the finding that phosphorylation and acetylation PTMs are more prevalent in stable proteins than unstable proteins. It is likely that the functional role of unstable proteins is regulated by their continual synthesis and degradation, while longer-lived proteins are regulated by modifications such as phosphorylation and acetylation. Indeed it has been suggested previously that combining a large-scale analysis of protein turnover with further PTM studies will shed insight onto key regulators of cellular responsiveness [5]. But there are variables other than PTMs that need to be explored in conjunction with protein stability. For example, the potentially stabilizing effect of complex formation, or cell- and tissue-specific degradation.

The benefit of applying a computational model is the ability to extend our knowledge beyond what is explicitly documented in experimental data. In this work we have used our model to create two sets of stability values for the human proteome – one using a model trained on all our data (P1), and one using a model trained on data cleansed from secreted and membrane proteins (P2). The model is able to generalise quite well, as seen through the ability to create new global stable and unstable protein classes that are consistent with the experimental data. Through analysis of these predicted values and classes, we were able to identify further features that are relevant for protein stability. For example, it appears that proteins containing NLS/NES domains are more likely to be stable than unstable. Similarly, our results are further evidence that protein modifications such as phosphorylation, acetylation and methylation are important regulators in protein degradation. We have also demonstrated how these predicted values, or the “stabilome” can be used to ask questions about the global stability of specific classes of proteins. As the predicted stability scores in P1 and P2 cover the human proteome and are made available in the Additional Files, interested readers can access the scores directly, obviating the need to use the predictor themselves.

A benefit of a computational model is its ability to be applied to different data sets. We have noted the disparity that currently exists between the major protein stability data sets (Table 1). We hypothesised that if the problematic secretory and transmembrane proteins were removed from the data set used here, the correlation would improve at least slightly with the other data, though that was not the case. Ideally we would have liked to test the model on a “blind” data set other than the GPSP data, but the lack of correlation between data sets brings into question the value of such a test. However, as more data becomes available, computational modeling of protein stability as applied here can be easily extended by retraining the model on new data. This will allow us to more effectively compare data sets and identify the common and distinct features that exist between them, to unravel the cell-specific and global features of the protein stabilome.

## Conclusions

New experimental techniques coupled with powerful data integration methods have enabled us to not only investigate what features govern protein stability in general, but also to build a model that identifies what properties determine each protein’s metabolic stability. This study shows how several post-translational modifications, domain types, N-terminal residues, disorder and sequence data can be incorporated into a model to classify proteins as stable or unstable as defined by experimental data. Bayesian networks are an ideal tool for such a task, with their ability to combine multiple forms of data and capture conditional dependencies that exist between features. At a 20% false positive rate, the model exhibits an 80% true positive rate, and outperforms the only previously proposed stability predictor. We have also considered the possibility of experimental bias within the data, retraining the model on a data set cleansed of secreted and transmembrane proteins. Computational models of protein stability are important not only for classifying proteins of unknown stability, but for comparing various experimental data sets. Furthermore, the use of the model to score all human proteins in the HPRD will be a freely available resource for researchers who are interested in the stability of functionally related proteins.

## Methods

### Data resources and feature identification

To identify discrete stability classes we used the normalised (values adding to one) 7-dimensional GPSP micro array data [1]. We grouped the proteins using the hierarchical clustering method provided in the standard R package. The method calculates Euclidean distances between vectors and groups the vectors with the closest distance in a pairwise manner, moving outwards to cluster pairs, and then groups, in a hierarchical manner (Figure 1). As we were interested in identifying dichotomous binary classes that could be used in training a classifier, we chose the two clusters at the top of the hierarchy that were grouped into stable and unstable proteins respectively.

To understand what specific PTMs and protein domain/architecture types correlate with measured protein stability, we used data from HPRD [30]. We also classified proteins according to the original N-end rule as described by Varshavsky and colleagues [12]. The beginning of the mature peptide is first predicted. To detect the location of signal peptide cleavage sites, we used the online predictor signalP (http://www.cbs.dtu.dk/services/SignalP). The protein set was further processed according to the N-end rule as described previously [2,31]. For proteins not containing a signal peptide, the initiating methionine residue was removed when the second residue was one of C, G, A, S, T, V or P. After processing, the new N-terminal residue was classified as destabilizing if either R, K, H, F, L, W, I or Y. Otherwise it was classified as stabilizing. We then compared the presence of destabilizing N-terminal residues between the stable and unstable protein groups.

An issue that needs to be considered is the bias potentially caused by the N-terminal tag in the data set generated by Yen and colleagues [1]. In order to create a subset of the data that was free from secreted and transmembrane proteins, to explore the possibility of such bias, we used the online predictors SignalP and TMHMM (http://www.cbs.dtu.dk/services/TMHMM/), respectively [32]. Approximately 30% of proteins were predicted to be either secreted or integral to the membrane. This figure is consistent with those previously reported in whole proteome analysis [33].

### Models for classifying protein stability

#### Classifying stability from sequence: SVM

We configured a SVM to accept as input the amino acid composition of a protein to classify it into either of two classes. In practice, the SVM will produce a score indicating the similarity between the input protein and the stable and unstable proteins in the training set (the so-called “support-vectors”). We used the 1-spectrum kernel developed by Leslie and colleagues [23] to map an amino acid sequence into a compositional vector. We analysed this simple SVM by mapping its output to a Boolean variable by fitting two Gaussian densities (one for stable and one for unstable proteins; Figure 2). To avoid over-fitting, the training samples were halved, with one half being used to train the SVM and the other half for finding the means and variances. The evaluation was performed on withheld test-samples, based on the class posterior probabilities: the probability of the class given the SVM score.

#### Classifying stability by integrating features: BN

The network structure of the BN+SVM model is shown graphically in Figure 2. The BN model is identical, but without the “Sequence SVM” node. The BN+SVM model incorporates the random variables, assigned values in accordance with the following observations of a query protein: 

1. Stability: Set to indicate whether the protein is stable (true) or unstable (false). The value is conditioned on the variables N-terms, PTM, Domain and Disorder described below.

2. N-terms: Set to indicate whether the protein has a stabilizing (true) or a destabilizing N-terminal residue (false).

3. PTM: Latent, non-observed Boolean node, conditioned on the status of four specific PTMs. The PTMs include the presence (true) or absence (false) of tyrosine phosphorylation, serine/threonine phosphorylation, acetylation or glycosylation. To alleviate issues with data scarcity, a noisy-OR assumption is made: all specific PTMs inhibit the PTM variable independently from one another.In the absence of information about actual phosphorylation, we also incorporate the maximum match score collected from the raw amino acid sequence. Based on a position-weight matrix (PWM) created from putative modification sites, this score is, similar to the SVM score, incorporated as a continuous variable into the BN, to assign a value to the Boolean phosphorylation variable (treating it as a latent variable).

4. Domain: Latent Boolean node, conditioned on the presence/absence of nine specific protein domain and architecture types (Tables 2 and 3 contains the full list of domains). As for the PTM node, a noisy-OR assumption is made.

5. Disorder: Latent Boolean node, conditioned on whether the protein contains 80-100% “Loops/Coils”, and whether it contains 80-100% “hotloops” disorder types as defined in section “Identifying features correlated with stability”.

6. Sequence SVM: Set according to the continuous score of the SVM (which is trained to discriminate between stable and unstable protein sequences – see Section “Classifying stability from sequence: SVM”). Two Gaussian densities allow the BN to determine a class probability for any score, conditioned on the Stability node above.

#### Data sets for training and testing

The data set used for training and testing the classifiers was chosen based on the availability of HPRD information for PTMs and domain/architecture. The resulting data set contains 1537 proteins, divided into 743 stable and 794 unstable proteins, each represented by 19 features (2442 proteins were non-assigned and used as background in the statistical analysis). After screening the set for secreted and transmembrane proteins, we used 300 of the remaining stable proteins and 227 unstable proteins.

#### Performance metrics

We calculated AUC, MCC, and sensitivity as described previously [27]. The remaining scores are defined as follows, where *TP* is the number of true positives, *FP* the number of false positives, *TN* the number of true negatives, and *FN* the number of false negatives.

Precision:

(3) $$P=\frac{\mathrm{TP}}{\mathrm{TP}+\mathrm{FP}}$$

Recall:

(4) $$R=\frac{\mathrm{TP}}{\mathrm{TP}+\mathrm{FN}}$$

F-score:

(5) $$F=2\times \frac{P\times R}{P+R}$$

Specificity:

(6) $$\mathrm{spec}=\frac{\mathrm{TN}}{\mathrm{TN}+\mathrm{FP}}$$

#### Predicting phosphorylation from amino acid sequence

Experimentally confirmed phosphorylation sequence motifs were downloaded from the HPRD phosphoMotif finder at http://www.hprd.org/PhosphoMotif_ﬁnder[34]. We also took sequence data for experimentally confirmed phosphorylation sites from the Eukaryotic Linear Motif (ELM) database at http://phospho.elm.eu.org[35]. We created two classes of PWMs for predicting (1) tyrosine phosphorylation and (2) serine/threonine phosphorylation. We used 132 tyrosine phosphorylation motifs and 107 serine/threonine phosphorylation motifs. To create the PWMs, each motif was scanned over known phosphorylation sites, and if a match was found, the sequence matching the motif was added to a foreground set for that motif.

Each amino acid in a PWM was calculated by the following equation:

(7) $$\mathrm{score}=\overset{n}{\underset{i=1}{\sum }}\log(\frac{F_{i}+P}{\mathrm{BG}})$$

where *n* is the number of sequences containing a motif, *F*_*i*_ is amino acid frequency in the foreground set, *BG* is the amino acid frequency in the background set and *P* is a pseudo count calculated as *BG*/10. The PWMs were trained using a subset of proteins from the ELM database that did not overlap with the protein set used for training the Bayesian network.

### Comparison with previous work

The performance of the SVM, BN and BN+SVM models was compared with the IFS plus NN method described by [7] and provided by the authors. We used a subset of proteins contained in our “stable” class that overlapped with the “extra long” class used by [7], as well as a subset of our “unstable” class that overlapped with their “short” class. When evaluating the performance of our models on this data subset we trained using the full data set (i.e. the proteins in our stable and unstable classes), but tested only using these overlapping subsets.

To evaluate the performance of the NN predictor, we used their set of optimal feature components and values for the “short/medium *vs* long/extra long” classifier. To produce a continuous output from NN for analysis by ROC, we used the following function: *score*=*D*^−^−*D*^ + ^, where for a given test sample, *D*^−^is equal to the minimum distance between the test sample and all negative training samples, and *D*^ + ^ is equal to the minimum distance between the test sample and all positive training samples. For our analysis, proteins in the “unstable/short” class were considered negative, while proteins in the “stable/extra long” class were considered positive.

## Competing interests

The authors declare that no competing interests exist.

## Authors’ contributions

Conceived and designed the experiments: RP, MB and KAL. Performed the experiments: RP and MB. Analyzed the data: RP, KAL, MD, BK, MB. Drafted the paper: RP. Contributed to the final version of the paper: RP, KAL, MD, BK, MB. All authors read and approved the final manuscript.

## Supplementary Material

Additional file 1: **Data Set 1.** We analysed data provided by Huang and colleagues [7] to determine what sub cellular locations are over- or under-represented in their “short” or “extra long” protein classes. Additional file 1: Data Set 1 contains the list of sub cellular locations that were found to have a statistically significant (Fisher’s exact test, E-value < 0.05) presence in one of these stability classes. Click here for file

Additional file 2: **Figure S1.** The BN+SVM model was trained on the full dataset and used to score all proteins contained in the HPRD (P1). Additional file 2: Figure S1 shows the density plot for the prediction scores contained in P1. The bimodal nature of the distribution is reflective of the model’s training on stable and unstable proteins. Click here for file

Additional file 3: **Figure S2.** All proteins in HPRD were also scored using the BN+SVM model trained on the trimmed data set (P2). Additional file 3: Figure S2 shows the density plot for the prediction scores contained in P2. Due to the smaller amount of training data, there were some observations that the Bayesian network had never “seen” before. As a result, those proteins were given a score of 1. Click here for file

Additional file 4: **Data Set 2.** With our proteome wide scoring of stability (P1 and P2), we created predicted stability classes named “stable”, “unstable” and “non-assigned” based on scoring thresholds. We then re-examined domain and architecture data to determine what features were over- or under-represented in the predicted stability classes (Fisher’s exact test, E-value < 1e-05). Additional file 4: Data Set 2 contains the list of over- and under-represented domain and architecture types. Click here for file

Additional file 5: **Data Set 3.** Using the predicted stability classes from P1 and P2, we also examined what PTMs were significantly over- or under-represented amongst predicted stable and unstable proteins (Fisher’s exact test, E-value < e-10). Additional file 5: Data Set 3 contains this list of PTMs. Click here for file

Additional file 6: **Data Set 4.** We trained the BN+SVM model on the both the full data set (Additional file 6: Data Set 6) and the trimmed data set (Additional file 5: Data Set 7), and used these two models to generate stability scores for all proteins in HPRD. Additional file 6: Data Set 4 contains these two sets of scores (P1 and P2). Click here for file

Additional file 7: **Data Set 5.** We trained two SVM models using the sequence data from Additional file 8: Data Set 6 and Additional file 9: Data Set 7, and used these models to generate SVM scores for all proteins in HPRD. Additional file 4: Data Set 5 contains these two sets of scores. Click here for file

Additional file 8: **Data Set 6.** Data Set 6 contains the full set of feature vectors used for training/testing. This data was also used for training the BN+SVM model to produce the P1 data set. The feature vectors contain PWM scores, Boolean values representing the presence of PTMs, domains, “destabilizing” N-terminal residues, disorder types and sequence data. Click here for file

Additional file 9: **Data Set 7.** Data Set 7 contains the trimmed set of training/testing feature vectors with secreted and transmembrane proteins removed. The feature vectors contain the same type of information as in Additional file 8: Data Set 6. This data was used for training the BN+SVM model to produce the P2 data set. Click here for file

Additional file 10: **Data Set 8.** Data Set 8 contains the full set of feature vectors for all proteins in HPRD with the same type of information as in Additional file 8: Data Set 6 and Additional file 9: Data Set 7. These feature vectors were evaluated by the classifier after being trained on Additional file 8: Data Set 6 and Additional file 9: Data Set 7 to produce, respectively, the P1 and P2 scores. Click here for file
