# Supplementary figures and images for: Mapping the stabilome: a novel computational method for classifying metabolic protein stability
Source: BMC Syst Biol. 2012 Jun 8;6:60. doi: 10.1186/1752-0509-6-60 (PMC3439251; doi:10.1186/1752-0509-6-60)

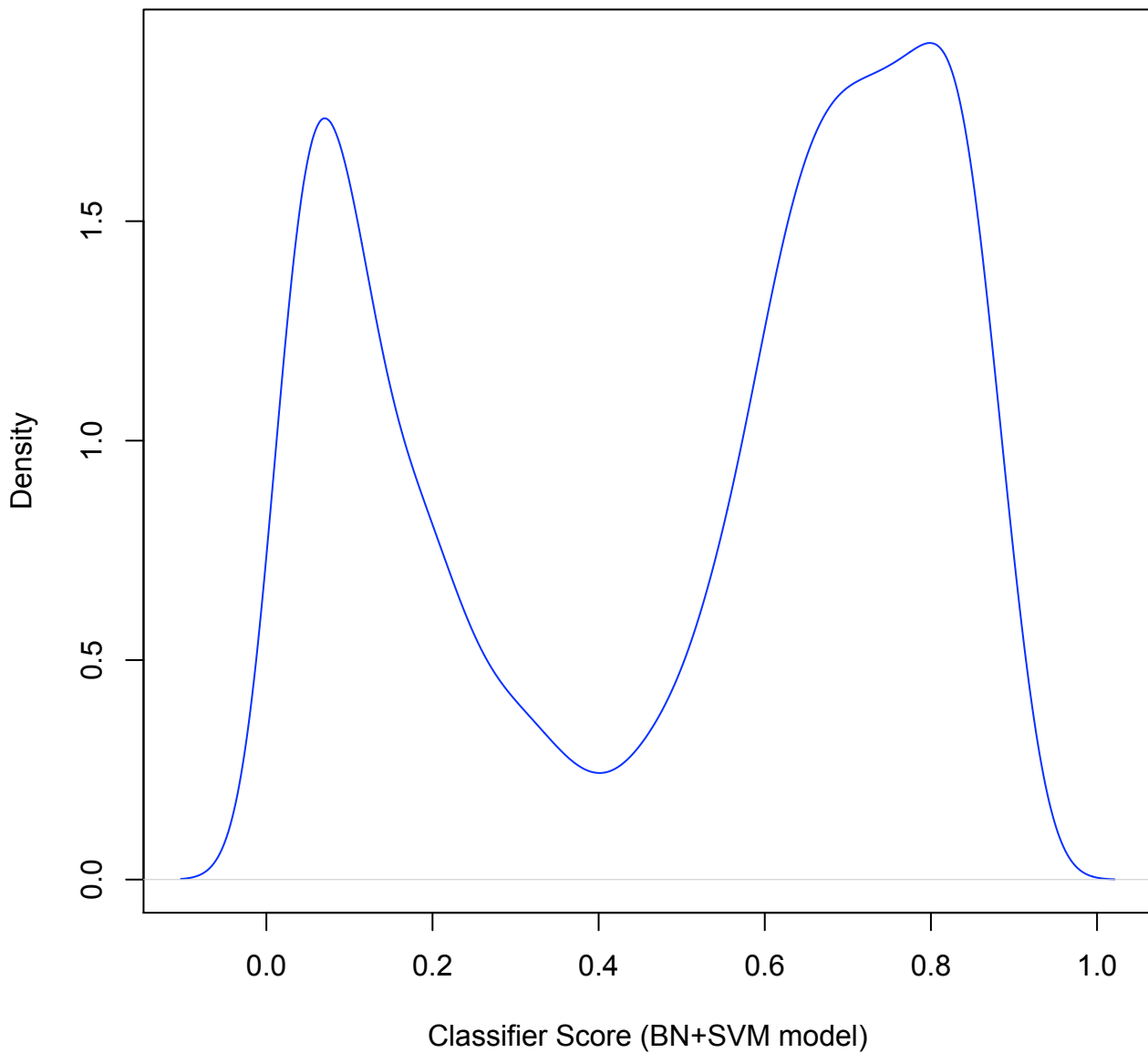

Supplement: Additional file 2: — Figure S1. The BN+SVM model was trained on the full dataset and used to score all proteins contained in the HPRD (P1). Additional file 2: Figure S1 shows the density plot for the prediction scores contained in P1. The bimodal nature of the distribution is reflective of the model’s training on stable and unstable proteins. [file 1752-0509-6-60-S2.pdf]

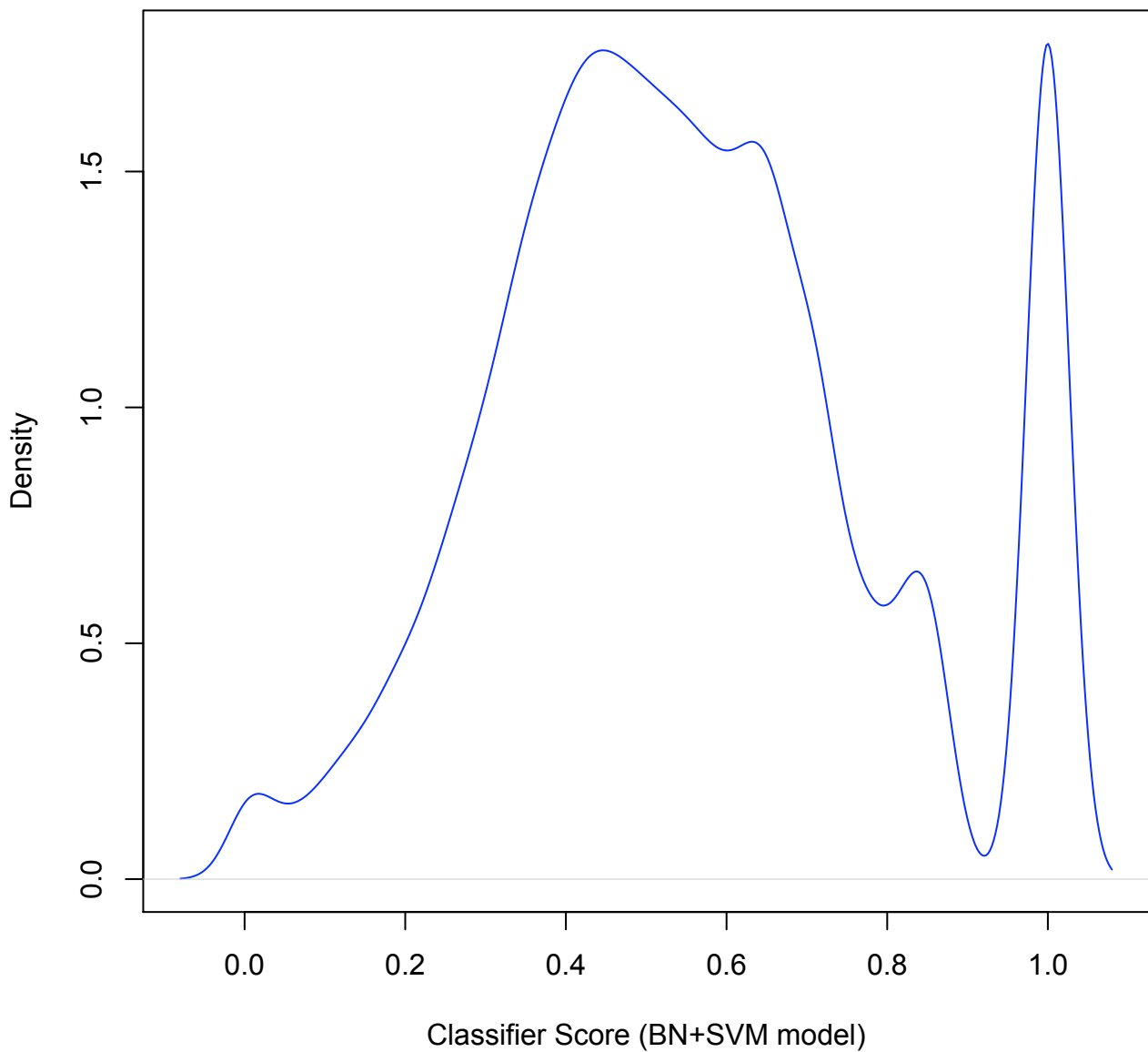

Supplement: Additional file 3: — Figure S2. All proteins in HPRD were also scored using the BN+SVM model trained on the trimmed data set (P2). Additional file 3: Figure S2 shows the density plot for the prediction scores contained in P2. Due to the smaller amount of training data, there were some observations that the Bayesian network had never “seen” before. As a result, those proteins were given a score of 1. [file 1752-0509-6-60-S3.pdf]
